# Isoforms of the Papillomavirus Major Capsid Protein Differ in Their Ability to Block Viral Spread and Tumor Formation

**DOI:** 10.3389/fimmu.2022.811094

**Published:** 2022-03-14

**Authors:** Daniel Hasche, Melinda Ahmels, Ilona Braspenning-Wesch, Sonja Stephan, Rui Cao, Gabriele Schmidt, Martin Müller, Frank Rösl

**Affiliations:** ^1^ Division of Viral Transformation Mechanisms, Research Program “Infection, Inflammation and Cancer”, German Cancer Research Center (DKFZ), Heidelberg, Germany; ^2^ Core Facility Unit Light Microscopy, German Cancer Research Center (DKFZ), Heidelberg, Germany; ^3^ Research Group Tumorvirus-specific Vaccination Strategies, Research Program “Infection, Inflammation and Cancer”, German Cancer Research Center (DKFZ), Heidelberg, Germany

**Keywords:** immune escape mechanism, vaccination, cutaneous papillomaviruses, *Mastomys coucha*, neutralizing antibodies, major capsid protein (L1), viral infection, NMSC (non-melanoma skin cancer)

## Abstract

Notably, the majority of papillomaviruses associated with a high cancer risk have the potential to translate different isoforms of the L1 major capsid protein. In an infection model, the cutaneous *Mastomys natalensis* papillomavirus (MnPV) circumvents the humoral immune response of its natural host by first expressing a 30 amino acid extended L1 isoform (L1_LONG_). Although inducing a robust seroconversion, the raised antibodies are not neutralizing *in vitro*. In contrast, neutralizing antibodies induced by the capsid-forming isoform (L1_SHORT_) appear delayed by several months. We now provide evidence that, although L1_LONG_ vaccination showed a strong seroconversion, these antibodies were not protective. As a consequence, virus-free animals subsequently infected with MnPV still accumulated high numbers of transcriptionally active viral genomes, ultimately leading to skin tumor formation. In contrast, vaccination with L1_SHORT_ was completely protective. This shows that papillomavirus L1_LONG_ expression is a unique strategy to escape from antiviral immune surveillance.

## Introduction

Mucosal human papillomaviruses (HPV) are the etiological agents for anogenital as well as head and neck tumors (1). Furthermore, many seroepidemiological and molecular studies revealed a decisive role of certain cutaneous HPV types in the development of non-melanoma skin cancer (NMSC), specifically squamous cell carcinomas (SCC) (2, 3). In this tumor entity, the viruses trigger carcinogenesis as co-factors in conjunction with chronic UV exposure *via* a hit-and-run mechanism (4). This indicates that viral presence is required for the initiation but not for the maintenance of the malignant phenotype. Taxonomically, these HPV types mainly belong to the beta and gamma genera and are ubiquitously present in the general population (5, 6). These commensal infections already occur in infancy (5, 7), can cause warts in young children (8) and usually remain asymptomatic in healthy adults. However, elevated antibody titers against cutaneous HPVs as well as high viral loads in eyebrow hair correlate with an increased risk of developing SCCs (9–11).

To achieve and maintain commensal infections in different species, PVs have developed various strategies to escape from natural innate and adaptive immune surveillance (12). To understand these mechanisms, the African multimammate rodent *Mastomys coucha* is used as a preclinical model. In these animals, the temporal and spatial spread of the cutaneous MnPV (*Mastomys natalensis* papillomavirus) can be investigated, starting from early infection until the final manifestation of a skin tumor in an immunocompetent host (13). Like humans, the animals become infected early after birth, which is accompanied by seroconversion against several viral antigens (14, 15). Notably, MnPV-infected *Mastomys* mimic many aspects of human skin carcinogenesis like the aforementioned cooperation of infection and UV exposure in SCC development *via* a hit-and-run mechanism (16). Immunization with MnPV virus-like-particles (VLPs) induces a long-lasting protection against skin lesions even under immunosuppressive conditions (17). Additionally, the availability of a virus-free *Mastomys coucha* colony allows testing potential vaccination strategies, since immunization efficacy can be challenged by subsequent experimental MnPV infection at defined time points and under standardized conditions (17).

Independently from the tissue tropism (either mucosal or cutaneous), a remarkable feature of many PV types causing serious clinical manifestations is the occurrence of alternative in-frame initiation codons (ATGs) upstream of the canonical L1 open reading frame (ORF; from here on referred to as L1_SHORT_), potentially allowing the translation of different isoforms of the major capsid protein (14). While only this L1_SHORT_ is capable to form genuine infectious virus particles and VLPs (14, 18), the biological function of other L1 isoforms and immune responses against them was hitherto unknown. Therefore, we recently measured time course and specificity of the humoral immune response in naturally MnPV-infected animals. Remarkably, seroconversion against the long L1 variant (L1_LONG_) preceded that against L1_SHORT_ by about four months, but only antibodies against the latter were neutralizing in *in vitro* assays and therefore protective (14). This time gap apparently enables successful MnPV infection and viral spread in the natural host, arguing for a humoral immune escape mechanism.

In the present study, we show that L1_LONG_ and L1_SHORT_-based vaccines completely differ in their ability to protect against experimental MnPV infection and subsequent skin tumor formation.

## Results

### Immunization With L1 Isoforms Induces Distinct Antibody Responses

**Figure 1:**
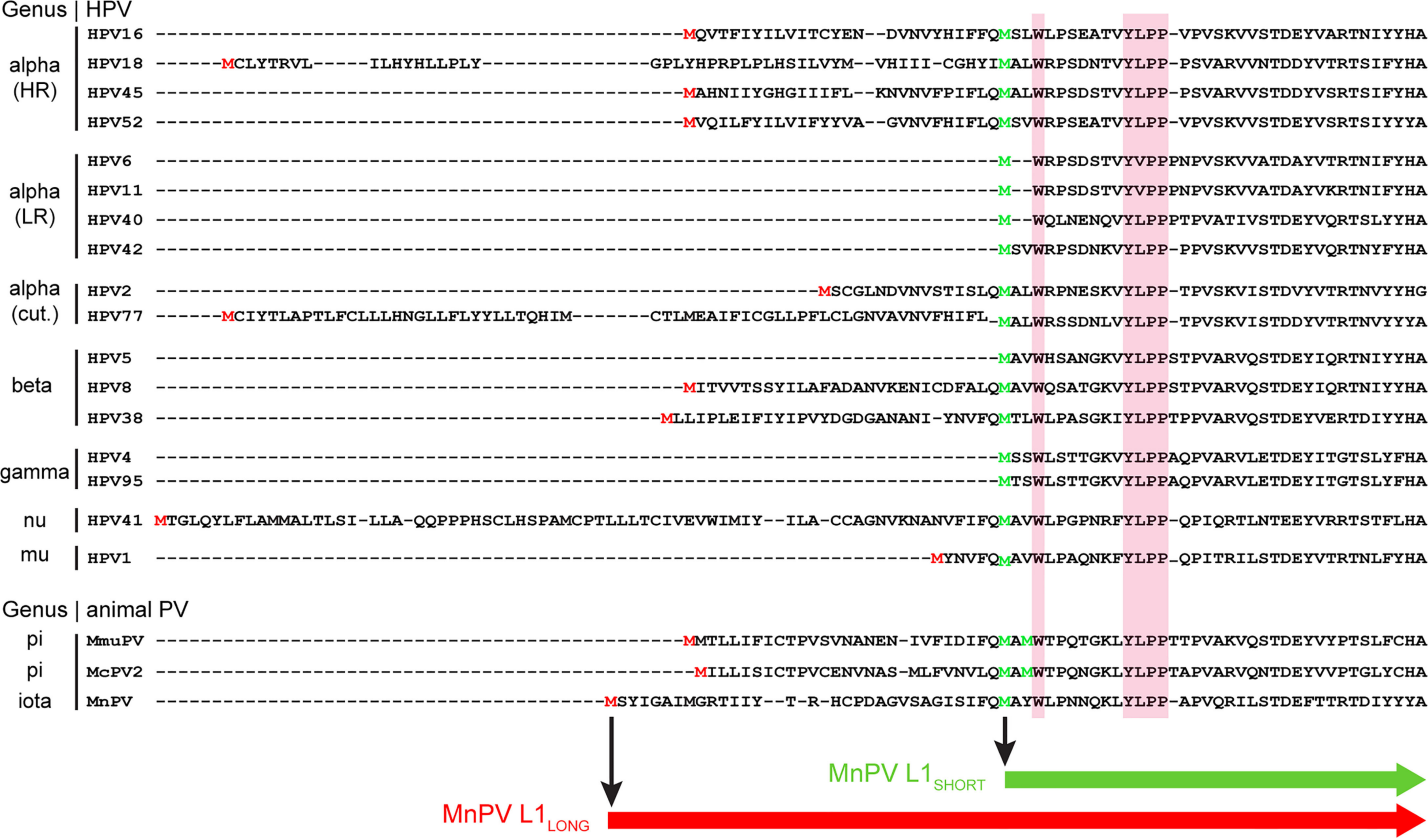
Localization of the MnPV L1 isoforms in comparison to other PV types. N-terminal amino acid sequences of L1 major capsid proteins from certain high-risk (HR), low-risk (LR) or cutaneous (cut.) HPVs and animal PV types of different genera were aligned using Clustal Omega. Pink boxes: highly conserved Wx7YLPP motif common to both human and animal PVs. Green M: methionine upstream of the Wx7YLPP motif initiates L1_SHORT_.Red M: upstream in-frame methionine of L1_LONG_. Figure modified from (14).

Due to the existence of alternative in-frame initiation codons within the MnPV L1 ORF, also present in various high-risk mucosal and cutaneous HPVs, different L1 isoforms could be potentially translated (**Figure 1**). To investigate whether the switch of L1 isoform expression during natural infection is the result of a humoral immune escape mechanism, we now addressed the question whether vaccination with different L1 isoforms confers protection against MnPV infection in an exploratory study.

**Figure 2:**
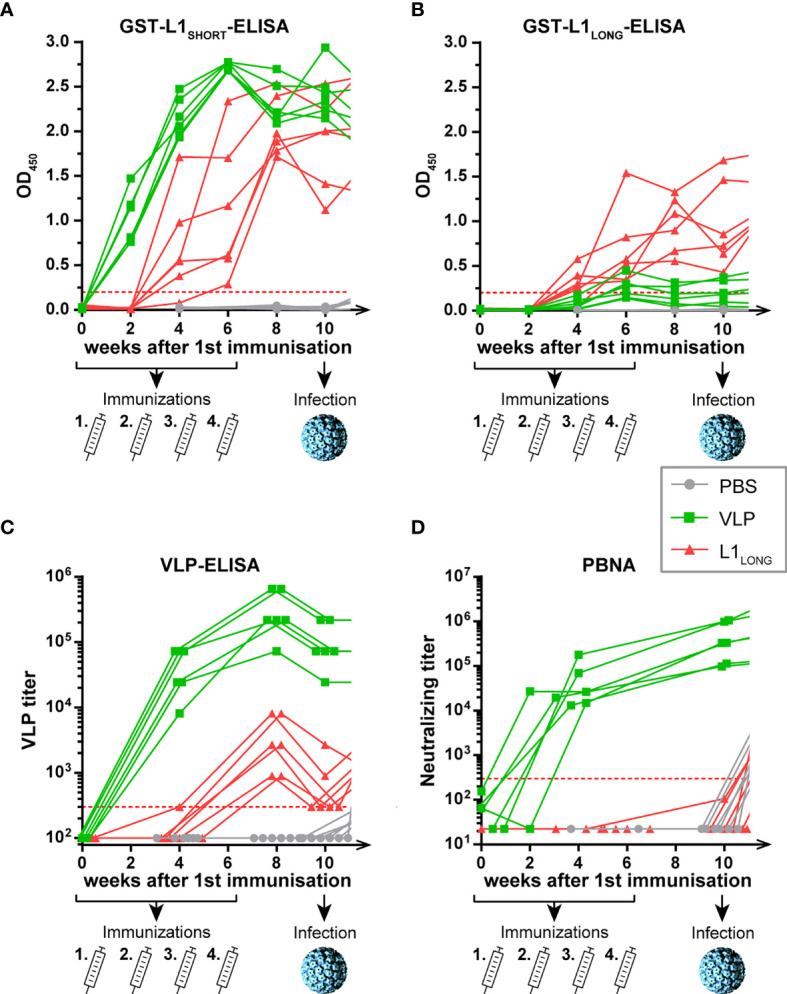
Seroconversion of L1_LONG_- and VLP-vaccinated animals. Seroresponses measured by GST-ELISAs against **(A)** L1_SHORT_, **(B)** L1_LONG_, **(C)** Virus-like-particles (VLP) and **(D)** in pseudovirion-based neutralization assay (PBNA). The animals were vaccinated four times with MnPV L1_SHORT_-derived VLPs (green), MnPV L1_LONG_ (red) or PBS (grey) in biweekly intervals from weeks 0 to 6 *prior to* an experimental infection at week 10. Number of animals for each group, n = 6. Dashed lines represent the methods’ cut-offs (OD_450_ = 0.2 for GST-ELISA or titer of 300 for VLP-ELISA and PBNA). Note that the graph is continued in **Figure 4**.

For that purpose, 8-week-old virus-free *Mastomys coucha* (six animals per group) were vaccinated four times in biweekly intervals until week 6, followed by experimental MnPV infection at week 10 (outlined in **Figure 2**) and followed for up to 52 weeks (continued in **Figure 4**). Since our previous study showed that kinetics of seroconversion and specificity of antibodies against L1_LONG_ and L1_MIDDLE_ variants were similar (14), we only compared the impact of L1_LONG_ and L1_SHORT_ vaccination on subsequent viral challenge and skin tumor formation.

No seroconversion was discerned in mock-vaccinated control animals (**Figures 2A, B**, grey lines), indicating that immune responses measured in L1_LONG_- and L1_SHORT_-GST-ELISAs were specific for the respective L1 antigens (**Figures 2A, B**, green and red lines). Conversely, all animals vaccinated with MnPV L1_SHORT_ that efficiently forms virus-like-particles (VLPs) showed a rapid and strong seroconversion already two weeks after the first administration. Although L1_LONG_ differs from L1_SHORT_ only by additional 30 N-terminal amino acids, sera from L1_SHORT_-VLP- and L1_LONG_-vaccinated animals showed distinct seroresponses in both GST-L1_LONG_- and VLP-ELISAs (**Figures 2B, C**). Indeed, in the L1_LONG_-GST-ELISA (**Figure 2B**), seroreactivities of L1_LONG_-vaccinated animals exceeded those of VLP-vaccinated animals. On the other hand, these sera reacted much weaker when tested in VLP-ELISA (**Figure 2C**) and their titers even declined to cut-off level at week 10, while the seroresponses of the VLP-vaccinated animals still remained high. Of note, and contrasting L1_SHORT_ vaccination, L1_LONG_ did not induce protecting antibodies as measured by pseudovirion-based neutralization assay (PBNA) (**Figure 2D**). These results show that antibodies induced by the two L1 isoforms have different neutralizing capacities, likely due to the extended N-terminus that interferes with the genuine self-assembly process.

### Reactivity Against L1_SHORT_ Correlates With Neutralizing Activity

**Figure 3:**
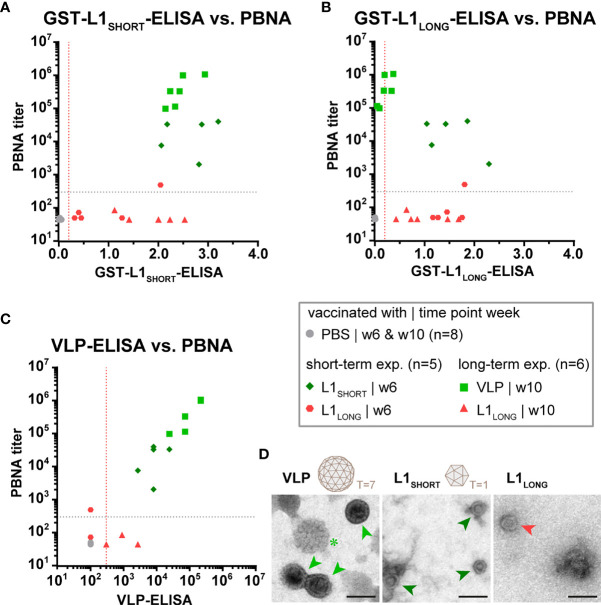
Only vaccination with L1_SHORT_ but not L1_LONG_ induces neutralizing antibodies. Correlation of data from **(A)** L1_SHORT_-GST-ELISA and PBNA, **(B)** L1_LONG_-GST-ELISA and PBNA, **(C)** VLP-ELISA and PBNA. Animals were vaccinated with MnPV VLPs (green squares, long-term experiment, n = 6 animals), MnPV L1_SHORT_ (dark green rhombus, short-term experiment, n = 5 animals see also **Supplementary Figure S1**), MnPV L1_LONG_ (red triangles, long-term experiment, n = 6 animals; red hexagons, short-term experiment, n = 5 animals, see also **Supplementary Figure S1**) or PBS (grey dots, long-term experiment, n = 6 animals). Dashed lines represent the methods’ cut-off (OD_450 _= 0.2 for GST-ELISA or titer of 300 for VLP-ELISA and PBNA). **(D)** Morphology of different L1 antigen preparations (VLPs, L1_SHORT_, L1_LONG_) by negative stain electron microscopy at 16,000x magnification (scale bar: 50 µm). Schemes depict 72-pentamer (T = 7) and 12-pentamer (T = 1) particles as previously described (19).

The distinct seroresponses after vaccination with different L1 isoforms became more obvious when ELISA and PBNA data were correlated (**Figure 3**). Here, independently of whether the animals were immunized either with L1_SHORT_-VLPs assembled in insect cells or with L1_SHORT_ produced in bacteria (see also **Supplementary Figure S1**), sera of animals vaccinated with L1_LONG_ or PBS cluster differently (correlation coefficients, see also **Supplementary Table S1**). This shows that L1_SHORT_ can form highly immunogenic VLPs and smaller structural entities irrespective of the source of their production (20). This notion was further substantiated by electron micrographs of the purified antigens used for this vaccination study (**Figure 3D**). In line with our previous results (14), highly pure and concentrated intact spherical T=7 VLPs of 50-60 nm diameter with clearly detectable capsomers could be visualized (**Figure 3D**, green asterisk). Since some VLPs were filled with uranyl during the preparation for EM, they appear darker in the negative stain (**Figure 3D**, light green arrows). EM examination of bacteria-derived L1_SHORT_ antigen revealed round particles with a size of approximately 25 nm diameter. They appear like T=1 icosahedrons made of 12 pentamers (**Figure 3D**, dark green arrows), reminiscent to VLPs made of N-terminally truncated HPV16 L1 (19). In contrast, monitoring the L1_LONG_ antigen, only a small number of structured spheres with structural similarity to the T=1 particles could be found (**Figure 3D**, red arrow). However, despite the presence of these spheres, antibodies induced by L1_LONG_ failed to neutralize infectious pseudovirions, a necessity to protect against viral infection.

### Antibodies Against L1_SHORT_ but Not Against L1_LONG_ Protect Against Viral Infection and Virus-Induced Skin Tumors *In Vivo*

**Figure 4:**
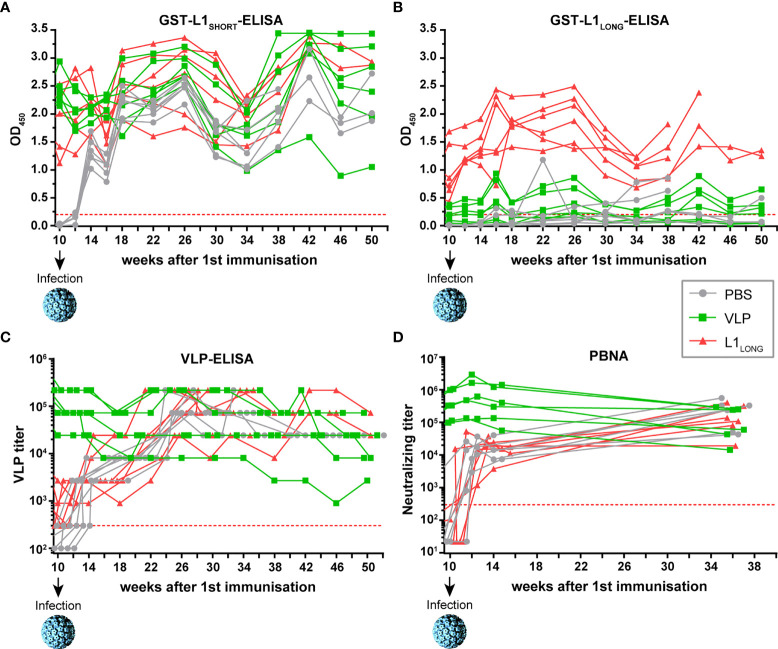
Monitoring seroresponses of vaccinated animals after viral challenge. Follow-up of seroresponses measured by GST-ELISA against **(A)** L1_SHORT_, **(B)** L1_LONG_, **(C)** VLPs and **(D)** in PBNA after experimental infection of vaccinated animals at week 10. Prior to infection, these animals were vaccinated with MnPV VLPs (green), MnPV L1_LONG_ (red) or PBS (grey) (for week 0 to 10, see **Figure 2**). All groups: n = 6 animals. Dashed lines represent the methods’ cut-off (OD_450 _= 0.2 for GST-ELISA or titer of 300 for VLP-ELISA and PBNA).

Next, we examined the course of the immune response against the L1 isoforms and the protective effect of vaccination after viral challenge. For this purpose, the animals were infected with MnPV particles from a papilloma extract at week ten (four weeks after the last immunization). As especially seen in the mock-vaccinated group, seroreactivity against L1_SHORT_ occurred already within four weeks after infection and continuously increased as detected by L1_SHORT_- and VLP-ELISAs (**Figures 4A, C**), accompanied by the appearance of neutralizing antibodies (**Figure 4D**). Likewise, sera of the L1_LONG_ group also showed an increase in reactivity determined by VLP-ELISA and PBNA, which remained stable over weeks (**Figure 4C**). Reactivity against L1_LONG_ in the corresponding GST-ELISA was not elevated after experimental infection (**Figure 4B**), which is consistent with previous experiments, showing low L1_LONG_ expression levels in tissue (14). Since VLP-vaccinated animals already showed high titers, experimental infection could not further elevate their reactivities in VLP-ELISA and PBNA (**Figures 4C, D**).

**Figure 5:**
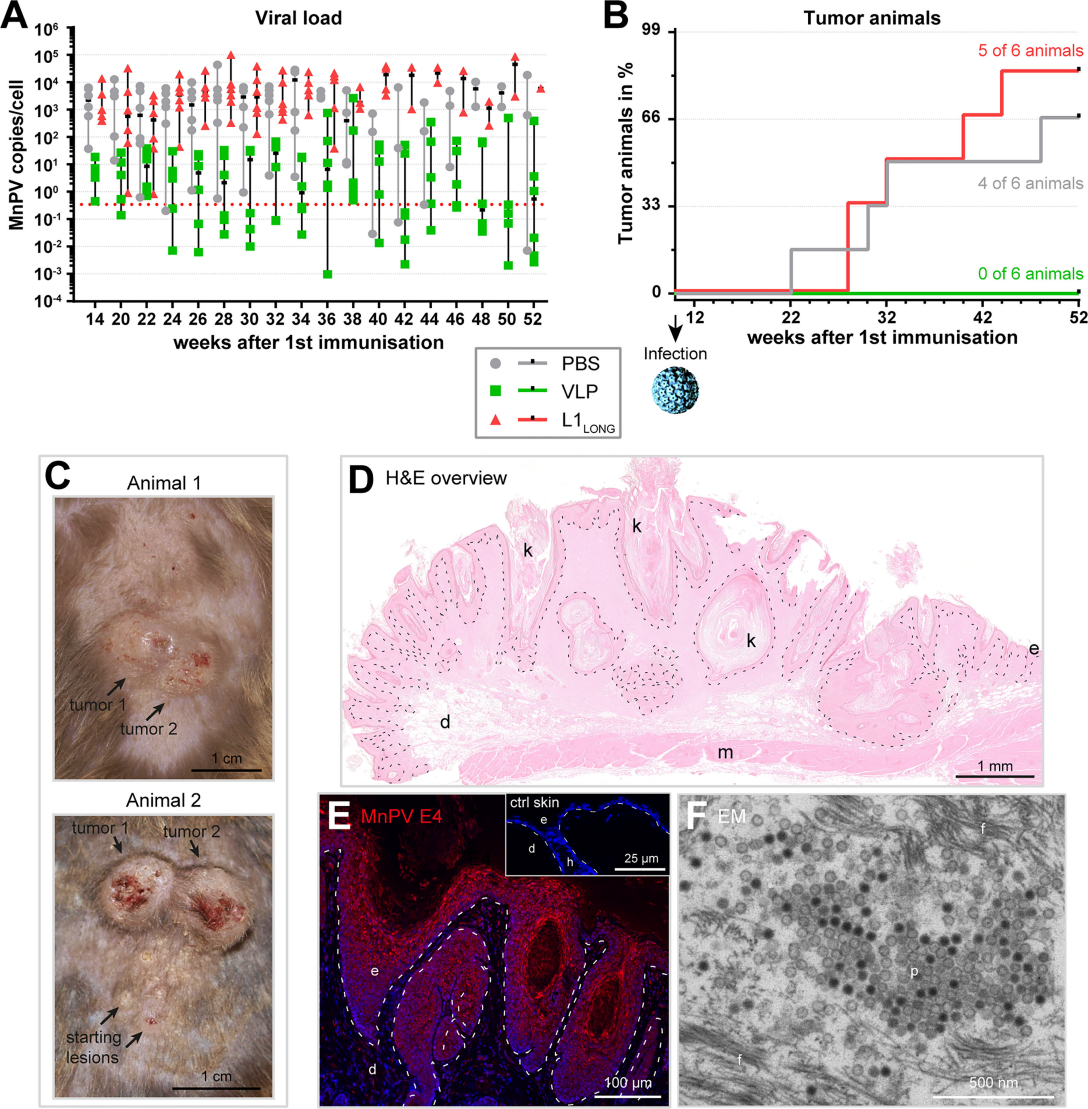
Vaccination with L1_LONG_ does not prevent viral accumulation and tumor formation after experimental infection. **(A)** The viral load of the vaccination groups was followed over time *via* qPCR of DNA extracted from plugged hair. The dashed line represents the methods’ cut-off based on hair extracted *prior to* the experimental infection (0.346 MnPV copies/cell). **(B)** Appearance of tumor-bearing animals in the different vaccination groups (median PBS group: 40 weeks, median L1_LONG_ group: 36 weeks; PBS *vs*. VLP: p = 0.0183; L1_LONG_ *vs*. VLP: p = 0.0041; log-Rank test). **(C)** Representative examples of MnPV-induced skin tumors after experimental infection. **(D)** H&E staining of a MnPV-induced papilloma (animal 1, tumor 2). **(E)** Immunofluorescence staining against the MnPV E4 protein in the epidermal part of same papilloma. The inset shows an E4 stained virus-free skin as negative control. **(F)** Electron micrograph of virus progeny located in the *stratum corneum* of a MnPV-induced papilloma (f: filaments, d: dermis, e: epidermis, h: hair follicle, k: keratinized material, m: muscle, p: particles).

To examine whether vaccination with L1_SHORT_-derived VLPs or L1_LONG_ can protect the animals against experimental MnPV infection at week 10 and subsequent tumor formation, the viral load in plugged hair bulbs was measured (**Figure 5A**). It took approximately 4 weeks until the hair started to re-grow after initial shaving and samples could be regularly collected. As depicted in **Figure 5A**, at week 14 the median viral loads were above the methods’ cut-off in all groups, indicating that experimental infection was successful. However, in both PBS- and L1_LONG_-vaccinated animals, the median increased several log-folds higher (to 100-10,000 copies/cell) in comparison to the VLP group (to 1-10 copies/cell) during the first ten weeks post infection. This unequivocally shows that only neutralizing antibodies raised by the L1_SHORT_-derived VLP vaccine but not the immunization with L1_LONG_ could protect the animals from MnPV infection.

The ultimate read-out when testing vaccinations against a tumor virus is the prevention of tumor formation (**Figure 5B**). Consistent with the high viral DNA loads in the PBS control and L1_LONG_ groups (**Figure 5A**), within the 52 weeks of observation, the number of skin tumor-bearing animals increased to four out of six (=66%) and five out of six animals (=83%), respectively. Conversely, all six VLP-vaccinated animals remained tumor-free (PBS vs. VLP: p=0.0183; L1_LONG_ *vs*. VLP: p=0.0041; log-Rank test) (**Figure 5B**).

The skin tumors (**Figures 5C, D**) resembled exo-endophytically growing papillomas and keratoacanthomas, also occurring on older MnPV-infected animals (16, 17). These skin tumors harbor transcriptionally active viral genomes and viral expression can be visualized by immunofluorescent staining of MnPV E4 (**Figure 5E**). The E4 protein already appears in suprabasal keratinocytes and accumulates towards the outer layers of the lesion (**Figure 5E**). In keratinized areas (*stratum corneum*) new virus progeny can be found (**Figure 5F**), indicating that the viral infection cycle is completed. Taken together, these data show that antibodies against the L1_LONG_ isoform, as they also arise during natural infection (14), are not protective against MnPV infection, even when induced by vaccination.

## Discussion

Several mucosal and cutaneous papillomaviruses of different genera harbor an in-frame ATG initiation codon upstream of the ORF encoding the L1 major capsid protein, potentially allowing the translation of N-terminally elongated L1 isoforms (**Figure 1**). Notably, these PV types are mostly associated with cancer (e.g. high-risk HPVs), while low-risk types such as HPV6 and 11 could not express putative alternative isoforms (21). Hence, there must be an evolutionary pressure to maintain additional L1 initiation codons, allowing the synthesis of longer isoforms to acquire a selective advantage of these oncogenic PVs types.

Using *Mastomys coucha* as a preclinical model, serological analyses revealed a temporally defined order of seroconversion against L1 isoforms during natural infection with the cutaneous MnPV (14). The appearance of neutralizing antibodies against the MnPV L1_SHORT_ variant was delayed, while the expression of longer L1 isoforms seems to circumvent immune surveillance during early infection. This might enable the virus to successfully replicate and to spread in its natural host (14).

It is well known that infection-elicited natural antibody titers are lower than antibody levels induced by HPV vaccines (22). Moreover, since there are still no robust criteria that define the antibody titers necessary for protection (23), one could presume that the immune response against the L1_LONG_ isoforms may be not high enough to provide sufficient protection during the natural course of a MnPV infection. To get insight into this question, we tested the efficacy of L1_LONG_ and L1_SHORT_ vaccines in virus-free animals for protection against subsequent viral challenge. This allowed us to study the relationship between neutralization of pseudovirions *in vitro* (24, 25) and the ultimate protection against skin tumor formation *in vivo.*

As presented in **Figures 2A–C** (see also **Supplementary Figure S1**), irrespective of whether L1_SHORT_, L1_LONG_ or VLPs were used for vaccination, an overall humoral immune response during the first six or eight weeks could be measured. However, comparing the results of the GST-L1_LONG_- and L1_SHORT_-ELISAs (**Figures 2A, B**), differences in reactivity became obvious. This can be explained by the fact that L1_LONG_ – although essentially consisting of L1_SHORT_ – is N-terminally extended by 30 amino acids (**Figure 1**). The extended N-terminus apparently sterically hinders a proper self-assembly to VLPs (19) as shown for L1_SHORT_ (**Figure 3D**). Concomitantly an additional conformational epitope is formed, which is lost under denaturizing conditions (14). Sera directed against L1_LONG_ were non-neutralizing even after several boosts with the L1_LONG_ vaccine (**Figures 2D**, **3B**). Even artificial modification of single residues within the HPV L1 protein affects VLP size and dramatically influences humoral immune responses against these particles (26).

Notably, the inability of bacterially expressed L1_LONG_ to induce neutralizing antibodies was not due to unrelated recombinant protein expression systems (insect cells *versus* bacteria), since animals vaccinated L1_SHORT_ synthesized in *E. coli* developed high neutralizing antibody titers (**Supplementary Figure S1**). Moreover, the use of a prokaryotic system for both L1_LONG_ and L1_SHORT_ excludes potential eukaryotic posttranslational modifications of the major capsid protein (27, 28) that may account for differences in isoform immunity. As already reported for HPV16 and 18 (19, 29), cleavage of the GST-L1 fusion protein also enables bacteria-derived MnPV L1_SHORT_ to self-assemble to highly immunogenic VLPs. Indeed, electron microscopic examination (**Figure 3D**) of bacterially produced L1_SHORT_ revealed substantial amounts of particles that were, although smaller, still as spherical as insect cell-derived VLPs. In contrast, monitoring the L1_LONG_ production, only few irregularly shaped entities could be visualized. This is also in line with our previous observations that L1_LONG_ cannot form correctly assembled VLPs in Sf9 insect cells or infectious pseudovirions even in the presence of L2 (14). Importantly, while intact VLPs activate dendritic cells, which play a critical role in inducing adaptive immunity and generation of high neutralizing antibody titers, assembly-defective L1 and predominantly disordered capsomers do not exert this function (30).

Experimental exposure of *Mastomys* skin to infectious MnPV particles only induced antibodies measurable in GST-L1_SHORT_ and VLP-ELISAs (**Figures 4A, C**), but not in GST-L1_LONG_-ELISA (**Figure 4B**). This was anticipated, since L1_LONG_ cannot be incorporated into mature virus particles (14). Conversely, the strong induction of neutralizing antibodies in both L1_LONG_ and PBS animals observed in VLP-ELISA and PBNA (**Figures 4C, D**) shows that the experimental infection essentially triggers the same serum response like a VLP-based vaccination. Indeed, VLPs can mainly function like HPV virions since both harbor highly repetitive structures (31, 32) that can induce B cell responses and neutralizing antibodies without CD4^+^ T helper cell involvement (33), which is the basis for the available protective VLP-based HPV vaccines.

To monitor the viral load over time, we established a simple and time-effective method for DNA extraction from plugged hair obtained from the back of the animals. As summarized in **Figure 5A**, vaccination with L1_SHORT_-based VLPs efficiently kept the median viral load in a range of 1-10 copies/cell, while the copy number was 100 to 1,000-fold higher in both PBS controls and L1_LONG_-vaccinated animals. This is consistent with the induction of high neutralizing antibody titers by vaccination with L1_SHORT_-derived VLPs that interfere with reinfection and viral spread.

Notably, several studies showed that the amount of HPV DNA in skin can be correlated with the risk of skin tumor formation, both in healthy and immunocompromised persons (34, 35). Our animal model represents exactly this scenario: only PBS- or L1_LONG-_vaccinated animals developed tumors, while animals vaccinated with L1_SHORT_-derived VLPs completely remained tumor-free (**Figure 5B**).

Histologically, these skin tumors can be characterized as papillomas and keratoacanthomas (**Figures 5C, D**), expressing high amounts of the viral MnPV E4 protein (**Figure 5E**) and representing a resource of virus progeny (**Figure 5F**). A prerequisite for a complete permissive cycle is the expression of the capsid-forming L1_SHORT_ isoform in immunologically privileged upper epithelial layers (**Figure 5F**) to avoid activation of Langerhans cells (36). Premature synthesis and self-assembly of L1_SHORT_ to highly immunogenic viral particles would be detrimental since an activation of the humoral immune response at this moment would counteract or even completely block virus progeny formation and viral spread. Synthesis of L1_LONG_ as a 30 amino acid extended isoform throughout the epithelium avoids such a scenario by circumventing the host immune response and interfering with the self-assembly process (14). Whether L1 isoforms have additional functions, for instance by interacting with E2 to enhance both transcription and replication as shown for HPV16 L1 (37), remains to be investigated. Finally, thinking in evolutionary terms it is reasonable to assume that L1 isoform expression apparently not only provides a selective advantage for MnPV but possibly also for high-risk HPVs that maintain additional initiation codons for translating their L1 major capsid protein.

## Materials and Methods

### Animals

Virus-free *Mastomys* were obtained from Janvier Labs (Le Genest-Saint-Isle, France). *Mastomys coucha* at the DKFZ were housed under SFP conditions in individually ventilated cages (Tecniplast GR900) at 22+/-2˚C and 55+/-10% relative humidity in a light/dark cycle of 14/10 h. *Mastomys* were fed with mouse breeding diet and allowed access to water *ad libitum*. According to the three R rules of animal experimentation, the animals used here were a subpart of a lager study also including testing the protective efficacy of novel cross-specific second generation anti-HPV vaccines in an exploratory study (n=6 animals per group; groups: PBS, MnPV L1_SHORT_-derived VLPs, MnPV L1_LONG_). However, since the context for the anti-HPV vaccines differs, this subpart will be published elsewhere.

### Antigen Preparation

L1_SHORT_-derived VLPs were produced in Sf9 insect cells and purified as recently described (14). L1_SHORT_ and L1_LONG_ were produced in 1-liter scale in *E. coli* BL21 rosetta as GST-L1-SV40tag fusion proteins as previously described (38). In case of L1_LONG_, the second and third ATGs were mutated (methionine to alanine) to exclusively guarantee L1_LONG_ expression. Briefly, after expression, bacteria were pelleted and resuspended in 10 ml equilibration/wash buffer (125 mM Tris-HCl, 150 mM NaCl, pH8.0) including 2 mM ATP and 1× cOmplete™, EDTA-free Protease Inhibitor Cocktail (Roche Diagnostics GmbH, Mannheim, Germany) for bacterial lysis. After sonication and centrifugation of the debris, the supernatant including the soluble GST-fusion proteins was loaded on a 3 ml Glutathione Spin Column from the Pierce™ GST Spin Purification Kit (Thermo Fisher Scientific, Rockford, IL, USA) and incubated for 1h at 4°C with over-head rotation. After elution of the lysate, unbound proteins were removed by several column washes with the equilibration/wash buffer included in the purification kit. To cleave the glutathione-bound GST-L1-SV40tag fusion protein at the thrombin cleavage site between GST and L1 (29), 15 U thrombin in 3 ml thrombin buffer (20 mM Tris-HCl, 150 mM NaCl, 2.5 mM CaCl_2_, pH8.0) were added and incubated with over-head rotation for 3h at 22°C. The L1-SV40tag was eluted from the column (elution was repeated with 2 ml equilibration/wash buffer), which retained the glutathione-bound GST. The eluate was washed, concentrated *via* 10K MWCO Protein Concentrator (Thermo Fisher Scientific, Rockford, IL, USA) and resuspended in PBS. The protein amount and purity were determined *via* Nanodrop2000 (Thermo Fisher Scientific Rockford, IL, USA) and Coomassie gel in comparison to known amounts of BSA.

### Vaccination and Experimental Infection

Animals were immunized at an age of 8 weeks and each group consisted of half males and females. VLPs were dialyzed against 50 mM Hepes, 0.3 M NaCl, pH7.4 and 10 µg VLPs were prepared with PBS and 50% Sigma Adjuvant System (SAS) (Sigma-Aldrich, St. Louis, MO, USA), containing monophosphoryl lipid A (MPL) and synthetic trehalose dicorynomycolate in squalene and Tween80 (17) as suggested by the manufacturer. For immunization with L1_SHORT_ and L1_LONG_, 15 µg antigen were prepared with PBS and 50% AddaVax (*In vivo*Gen, San Diego, CA, USA), a squalene-based oil-in-water nano-emulsion, as suggested by the manufacturer. The control group was injected with PBS and 50% AddaVax only. For all antigens, a volume of 150 µl was injected subcutaneously in a skin fold of the neck.

Animals were vaccinated three or four times (short-term or long-term experiment, respectively) in a biweekly schedule and artificially infected two weeks later (long-term experiment). Infection was performed at the shaved back of anaesthetized animals (3% isoflurane) with 30 µl mashed papilloma extract (containing infectious MnPV virions) that was obtained from a MnPV-induced papilloma from a previous study (17).

Blood was taken in intervals from two to eight weeks by puncturing the submandibular vein of anaesthetized animals, starting at the age of eight weeks. For the follow-up experiment, animals were monitored for the duration of their lifetime until they had to be sacrificed due to tumor development or decrepitude.

### GST-Capture ELISA

The GST-capture ELISA was performed as recently described (14). Briefly, 96well PolySorb ELISA plates (Thermo Fisher Scientific, Rockford, IL, USA) were coated overnight at 4°C with glutathione-casein diluted in carbonate buffer (pH9.6). The next day, the plate was blocked for 1 h at 37°C with casein blocking buffer (CBB, 0.2% casein in PBS-T: 0.05% Tween-20 in PBS) and then incubated with the respective antigen (bacterial lysate containing GST-antigen-SV40-tag fusion protein) for 1 h. To remove unspecific reaction against bacterial proteins or the GST-SV40-tag fusion protein, *Mastomys* sera were diluted 1:50 in CBB containing GST-SV40-tag and pre-incubated for 1 h. ELISA plates were washed four times with PBS-T and pre-incubated sera was added. After 1 h, plates were washed four times and HPR-conjugated goat anti-mouse IgG (H+L) antibody (1:10,000 in CBB, Promega GmbH, Walldorf, Germany) was applied for 1 h. Antibodies were quantified colorimetrically by incubating with 100 μl/well substrate buffer for 8 min (0.1 mg/ml tetramethylbenzidine and 0.006% H_2_O_2_ in 100 mM sodium acetate, pH6.0). The enzymatic reaction was stopped with 50 μl/well 1 M sulfuric acid. The absorption was measured at 450 nm in a microplate reader (Labsystems Multiskan, Thermo Fisher Scientific, Rockford, IL, USA). To calculate the serum reactivity against the respective antigen, sera were tested in parallel against the GST-SV40-tag fusion protein and the reactivity was subtracted from the reactivity against the GST-antigen-SV40-tag. Each ELISA was performed in duplicates at least. The cut-offs were calculated individually for each antigen by measuring sera of virus-free animals.

### VLP-ELISA

VLP-ELISAs were performed as previously described (17). Briefly, 96well PolySorb ELISA plates (Thermo Fisher Scientific, Rockford, IL, USA) were coated with 100 ng/well purified high quality L1_SHORT_-VLPs in 50 mM carbonate buffer pH9.6. The next day, plates were blocked with CBB and incubated for 1 h with three-fold dilutions of *Mastomys* sera in CBB. Then, plates were washed four times with PBS-T and incubated with goat anti-mouse IgG-HRP (1:10,000 in CBB, Promega GmbH, Walldorf, Germany). After four washes, color development and measurement were performed as described for the GST-ELISA. Antibody titer represents the last reciprocal serum dilution above the blank.

### Pseudovirion-Based Neutralization Assay (PBNA)

Animal sera (tested in duplicates) were diluted in medium and subjected to 1:3 serial dilutions in 96well cell culture plates (Greiner Bio-One GmbH, Frickenhausen, Germany) as previously described (24). Afterwards, 60 µl of diluted sera were mixed with 40 µl of pseudovirions (harboring a reporter plasmid encoding *Gaussia* luciferase) and incubated for 15 min at RT. Next, 50 µl of 2.5×10^5^ HeLaT cells/ml were seeded onto the pseudovirion-serum mixture and cultured for 48 h at 37°C. The activity of secreted Gaussia luciferase was measured 15 min after adding coelenterazine substrate and Gaussia glow juice (PJK Biotech, Kleinblittersdorf, Germany) according to the manufacturer’s instructions in a microplate luminescence reader (Synergy 2, BioTek Instruments, Inc, Winooski, VT, USA). The neutralization titer represents the reciprocal of the highest dilution that reduces the signal by at least 50%.

### Determination of Viral Load

Hair samples re-grew four weeks after experimental infection and could be plugged bi-weekly with clean forceps, yielding in approximately 100 hair roots from three random positions within the infected area for each time point. DNA was extracted *via* Chelex resin-based method where the hair was digested overnight in 150 µl Chelex resin (5% w/v in water; 100 - 200 mesh; Bio-Rad, Hercules, CA, USA) with 2 µg proteinase K in a ThermoMixer (Eppendorf, Hamburg, Germany) at 56°C and 300 rpm. Afterwards, the suspension was vortexed for 10 sec, heated at 99°C for exactly 8 min in a ThermoMixer, vortexed again for 10 sec and centrifuged at room temperature for 3 min at 12,000×g to pellet the Chelex resin. The supernatant was transferred into a new tube and stored at 4°C (short-term) or -20°C (long-term).

The qPCR was performed with 1 µl DNA per reaction using the iTaq Universal SYBR Green Supermix (Bio-Rad, Hercules, CA, USA), including 0,17 µg/µl BSA (New England BioLabs, Frankfurt am Main, Germany) and 0,5 µM forward/reverse primers for the MnPV L1 gene or the single-copy-number gene β-Globin to determine the number of input cell equivalents (17). Per reaction, MnPV DNA copy numbers were determined in duplicate by using standard curves generated in the same PCR run with a standard containing MnPV and β-globin plasmids. MnPV DNA load was defined as the number of MnPV genomes per two β-globin copies. The method cut-off of 0.346 copies/cell (Median + 3×SD) was calculated from median viral load and standard deviation (0.003 ± 0,114) using hair samples from 50 animals of the virus-free colony.

### Immunohistochemistry (IHC)

Staining of formalin-fixed, paraffin-embedded tumors was performed as previously described (16). Briefly, deparaffinized sections were subjected to heat-induced epitope retrieval (citrate buffer pH6.0), blocked with 5% goat serum in PBS and incubated with a self-made anti-MnPV E4 mouse monoclonal antibody overnight at 4°C. After washing, slides were incubated with Alexa594-conjugated anti-mouse antibody (Invitrogen, Carlsbad, CA, USA) and nuclei were stained with DAPI. Sections were mounted with Dako Faramount Aqueous Mounting Medium (Dako North America, Inc, CA, USA) and imaged with a Keyence BZ-9000 Microscope (Keyence Deutschland GmbH, Neu-Isenburg, Germany) or a Hamamatsu NanoZoomer S60 (Hamamatsu, Hamamatsu City, Japan).

### Electron Microscopy (EM)

L1 isoform preparations were fixed with buffered aldehyde solution (2% formaldehyde, 2% glutaraldehyde, 1 mM MgCl_2_, 2% sucrose in 100 mM calcium cacodylate, pH7.2), followed by post-fixation in buffered 1% OsO_4_, graded dehydration with ethanol and resin-embedding in epoxide (12 g glycid ether, 6.5 g NMA, 6.5 g DDSA, 400 μl DMP30, all from Serva, Heidelberg, Germany). Ultrathin sections at nominal thickness 60 nm and contrast-stained with lead-citrate and Uranylacetate were observed in a Zeiss EM 910 at 100 kV (Carl Zeiss, Oberkochen, Germany) and micrographs were taken with image-plates, scanned at 30 µm resolution (Ditabis micron, Pforzheim, Germany).

### Alignments

Papillomavirus L1 sequences were taken from PaVE (Papillomavirus Episteme; pave.niaid.nih.gov) (39) and aligned using Clustal 2.0.12

### Statistical Analysis

Data analysis and graphic representation were performed with GraphPad Prism 6.0 Software. Tumor development was calculated with the log-Rank test at 95% confidence interval and a p-value of 0.05 to assess significance.

## Data Availability Statement

The original contributions presented in the study are included in the article/**Supplementary Material**. Further inquiries can be directed to the corresponding authors.

## Ethics Statement

The animals are housed and handled in accordance with local (DKFZ), German and European statutes. All animal experiments were reviewed and approved by responsible Animal Ethics Committee for the use and care of live animals (Regional Council of Karlsruhe, Germany, File No 35-9185.81/G289/15 and 35-9185.81/G65/21).

## Author Contributions

Conceptualization: DH and FR. Methodology: DH, FR, and RC. Investigation: DH, SS, IB-W, and MA. Data curation: DH. Formal analysis: DH. Visualization: DH. Resources: FR, RC, and GS. Funding acquisition: DH and FR. Project administration: DH. Supervision: DH. Writing – original draft: DH, FR, and MM. Writing – revision: DH, FR. All authors contributed to the article and approved the submitted version.

## Funding

IB-W was funded by the Wilhelm Sander-Stiftung (grant number 2018.093.1 to DH). MA was funded by the German-Israeli Cooperation in Cancer Research (DKFZ-MOST), project number CA182 to FR. RC was supported by the China Scholarship Council (CSC) and Infect-ERA III, collaboration project HPV-MOTIVA (grant number 031L0095B to FR).

## Conflict of Interest

Author RC is currently employed by Retrolead (Shanghai) Biopharma Co., Ltd.

The remaining authors declare that the research was conducted in the absence of any commercial or financial relationships that could be construed as a potential conflict of interest.

## Publisher’s Note

All claims expressed in this article are solely those of the authors and do not necessarily represent those of their affiliated organizations, or those of the publisher, the editors and the reviewers. Any product that may be evaluated in this article, or claim that may be made by its manufacturer, is not guaranteed or endorsed by the publisher.

## Supplementary Material

Supplementary Figure S1 Seroconversion after vaccination with *E. coli*-derived L1_LONG_ and L1_SHORT_. Seroresponses of animals vaccinated three times with MnPV L1_SHORT_ (dark green), MnPV L1_LONG_ (red) or PBS (grey) were measured by ELISAs against **(A)** L1_SHORT_, **(B)** L1_LONG_, **(C)** VLP and **(D)** PBNA. PBS group: n=2 animals, L1 groups: n=5 animals. Dashed lines represent the methods’ cut-off (OD_450_ = 0.2 for GST-ELISA or titer of 300 for VLP-ELISA and PBNA).
