# Supplementary figures and images for: Isoforms of the Papillomavirus Major Capsid Protein Differ in Their Ability to Block Viral Spread and Tumor Formation
Source: Front Immunol. 2022 Mar 14;13:811094. doi: 10.3389/fimmu.2022.811094 (PMC8964102; doi:10.3389/fimmu.2022.811094)

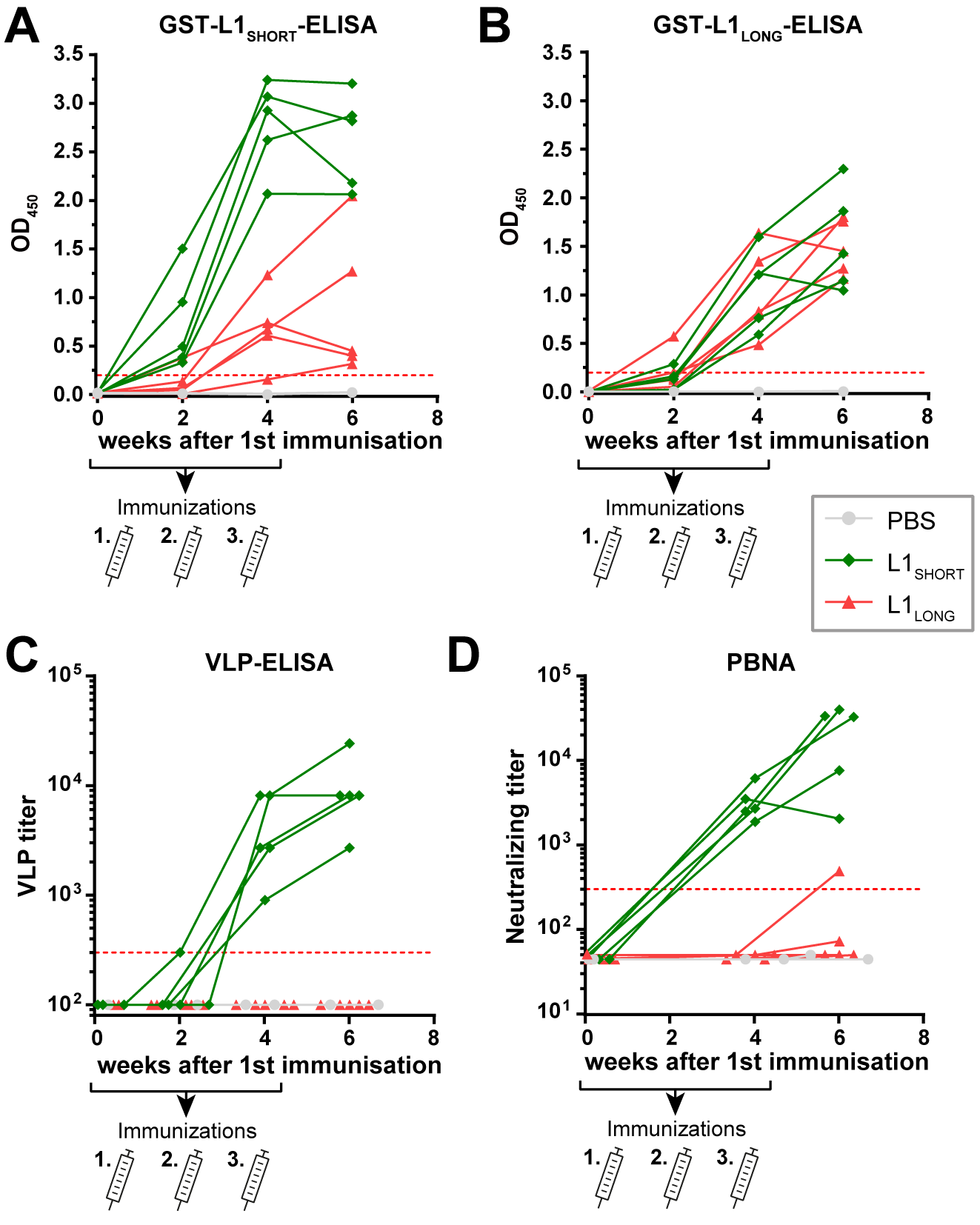

Supplement: Supplementary Figure S1 — Seroconversion after vaccination with E. coli-derived L1LONG and L1SHORT. Seroresponses of animals vaccinated three times with MnPV L1SHORT (dark green), MnPV L1LONG (red) or PBS (grey) were measured by ELISAs against (A) L1SHORT, (B) L1LONG, (C) VLP and (D) PBNA. PBS group: n=2 animals, L1 groups: n=5 animals. Dashed lines represent the methods’ cut-off (OD450 = 0.2 for GST-ELISA or titer of 300 for VLP-ELISA and PBNA). [file Image_1.tif]
