# Supplementary material for: Isoforms of the Papillomavirus Major Capsid Protein Differ in Their Ability to Block Viral Spread and Tumor Formation
Source: Front Immunol. 2022 Mar 14;13:811094. doi: 10.3389/fimmu.2022.811094 (PMC8964102; doi:10.3389/fimmu.2022.811094)
Supplement: Supplementary file 2 [file Table_1.docx]

**Supplemental Table S1: Correlation Coefficients for Fig 3.**

| **A)** Non-linear fit of correlation (log-linear axes) for Fig. 3A | | | | | |
| --- | --- | --- | --- | --- | --- |
| **Vaccination \| w6** | **Corr. Coeff.** | **Vaccination \| w10** | **Corr. Coeff.** | **Vaccination \|**  **w6 & w10** | **Corr. Coeff.** |
| - | | | | PBS | 0.01864 |
| L1_SHORT_ | 0.16300 | L1_SHORT_ | 0.61600 | L1_SHORT_ | 0.02164 |
| L1_LONG_ | 0.94860 | L1_LONG_ | 0.59260 | L1_LONG_ | 0.03284 |
|  | | | | | |
| **B)** Non-linear fit of correlation (log-linear axes) for Fig. 3B | | | | | |
| **Vaccination \| w6** | **Corr. Coeff.** | **Vaccination \| w10** | **Corr. Coeff.** | **Vaccination \|**  **w6 & w10** | **Corr. Coeff.** |
| - | | | | PBS | 0.00000 |
| L1_SHORT_ | 0.06649 | L1_SHORT_ | 0.36610 | L1_SHORT_ | 0.22610 |
| L1_LONG_ | 0.93260 | L1_LONG_ | 0.10270 | L1_LONG_ | 0.85840 |
|  | | | | | |
| **C)** Non-linear fit of correlation (log-log axes) for Fig. 3C | | | | | |
| **Vaccination \| w6** | **Corr. Coeff.** | **Vaccination \| w10** | **Corr. Coeff.** | **Vaccination \|**  **w6 & w10** | **Corr. Coeff.** |
| - | | | | PBS | 0.00000 |
| L1_SHORT_ | 0.24850 | L1_SHORT_ | 0.96230 | L1_SHORT_ | 0.97560 |
| L1_LONG_ | 0.00000 | L1_LONG_ | 0.07137 | L1_LONG_ | 0.11470 |
